# Supplementary material for: Gut Microbiota-Derived TMAO: A Causal Factor Promoting Atherosclerotic Cardiovascular Disease?
Source: Int J Mol Sci. 2023 Jan 18;24(3):1940. doi: 10.3390/ijms24031940 (PMC9916030; doi:10.3390/ijms24031940)
Supplement: Supplementary file 1 [file ijms-24-01940-s001.zip › ijms-2147558-supplementary.pdf]

International Journal of
Molecular Sciences

MDPI

Table S1. Additional Prospective studies about TMAO and mortality and other related cardiovascular outcomes.

Year	N	Follow-Up	Outcomes	Covariates in the Adjusted Models	HR (95% CI) and p Value	Ref.
			Acute or stable HF patients			
2014	720	5 years	All-cause mortality	Age, sex, systolic blood pressure, LDL-C, HDL-C, smoking, diabetes mellitus, and BNP<br>* + eGFR	UA: 3.42 (2.24–5.23) p < 0.001<br>Adj: 1.18 (1.06–1.31) p < 0.01<br>Adj*: 1.75 (1.07–2.86) p < 0.05	[55]
2015	155	5.2 years	All-cause mortality	NYHA class, age, hypertension, type 2 diabetes, HF etiology, eGFR, CRP, and NT-proBNP	UA: 2.24 (1.28–3.92) p = 0.005<br>Adj: 1.79 (0.90–1.79) p = 0.097	[83]
2015	112	5 years	All-cause mortality and cardiac transplantation	Age, eGFR, mitral septal E/Ea, and NT-proBNP	UA: 1.48 (1.10-1.96) p = 0.010<br>Adj: 1.46 (1.03-2.14) p = 0.031	[84]
2019	972	1 year<br>2 years<br>3 years	All-cause mortality<br><br>Death/HF	1: Age, blood urea, BNP, hemoglobin, and use of beta-blockers at baseline<br>2: model 1 + ischemic etiology, COPD, diastolic blood pressure, and sodium<br><br>1: age, previous HF hospitalization, peripheral edema, systolic blood pressure, BNP (log-transformed), hemoglobin, sodium, and use of beta-blockers at baseline<br>2: model 1 + current smoker, COPD, and eGFR	UA: 2.43 (1.93–3.05) p < 0.001<br>1: 1.51 (1.14–2.00) p = 0.004<br>2: 1.40 (1.03–1.89) p = 0.030<br>UA: 2.29 (1.90-2.77) p<0.001<br>1: 1.49 (1.18–1.88) p = 0.001<br>2: 1.44 (1.12–1.84) p = 0.004<br>UA: 2.27 (1.90–2.72) p < 0.001<br>1: 1.47 (1.18–1.84) p = 0.001<br>2: 1.42 (1.13–1.80) p = 0.003<br><br>UA: 1.92 (1.61–2.28) p<0.001<br>1: 1.42 (1.06–1.90) p = 0.019<br>2: 1.12 (0.90–1.40) p = 0.281<br>UA: 1.91 (1.64–2.22) p < 0.001<br>1: 1.39 (1.09–	[85]

					1.76) p = 0.007<br>2: 1.19 (0.98–1.45) p = 0.077<br>UA: 1.93 (1.66–2.23) p < 0.001<br>1: 1.37 (1.09–1.72) p = 0.007<br>2: 1.21 (1.00–1.46) p = 0.054	
2021	806	1 year	All-cause mortality<br><br>Death/HF	Sex, age, previous medical history HF, ischemic heart disease, hypertension, and diabetes mellitus	Adj: 1.26 (1.08–1.47) p = 0.004<br><br>Adj: 1.25 (1.09–1.42) p = 0.001	[86]
			T2D patients			
2017	1216	3 years<br><br>5 years	MACE<br><br>All-cause mortality	Age, sex, history of CVD, SBP, LDL-C, HDL-C, smoking, hsCRP, HbA1c, eGFR, BMI, and history of HF	UA: 3.03 (2.08–4.42) p < 0.001<br>Adj: 1.94 (1.23–3.05) p < 0.01<br><br>UA: 3.63 (2.53–5.21) p < 0.001<br>Adj: 1.85 (1.21–2.84) p < 0.01	[87]
2020	1463	7 years	MACE<br><br>All-cause mortality	Sex, age, and personal history of MI, eGFR, urine albumin-to-creatinine ratio, and NT-proBNP<br><br>Sex, age, sinus rhythm, SBP, ANGPTL2, and sTNFR1	UA: 2.91 (2.18–3.89) p < 0.001<br>Adj: 1.32 (1.02–1.70) p = 0.032<br><br>UA: 2.52 (1.98–3.21) p < 0.001<br>Adj: 1.16 (0.95–1.42) p = 0.151	[88]
2021	311	6.5 years	All-cause mortality<br><br>Cardiovascular mortality<br><br>CVD	Age, sex, HbA1c, systolic blood pressure, body mass index, total cholesterol, smoking, UAER, and eGFR	UA: 1.06 (0.88–1.28) p = 0.53<br>Adj: 1.02 (0.83–1.26) p = 0.85<br><br>UA: 1.09 (0.82–1.45) p = 0.54<br>Adj: 0.98 (0.70–1.37) p = 0.92<br><br>UA: 1.14 (0.96–1.35) p = 0.14<br>Adj: 1.11 (0.93–1.33) p = 0.26	[89]
2022	1468	7.3 years	Cardiovascular mortality	Age, sex, eGFR, and uACR	UA: 1.20 (1.10–1.31) p < 0.0001<br>Adj: NOT	[90]

					SIGNIFICANT	
			PAD patients			
2016	935	5 years	All-cause mortality	1: age, sex, systolic blood pressure, low-density lipoprotein cholesterol, high-density lipoprotein cholesterol, smoking, and diabetes mellitus<br>2: model 1 + hsCRP and eGFR<br>3: model 2 + history of CAD, statin use, apolipoprotein A1, apolipoprotein B and myeloperoxidase	UA: 2.69 (1.82-3.97) p<0.001<br>1: 2.06 (1.36–3.11) p<0.001<br>2: 1.59 (1.03–2.45) p<0.05<br>3: 1.88 (1.21–2.92) p<0.001	[91]
2019	262	4 years	All-cause mortality<br><br>Cardiovascular mortality	1: sex, age, smoking, diabetes mellitus, hypertension, dyslipidemia, HDL-C, eGFR<br><br>1: sex, age, and hs-CRP<br>2: diabetes mellitus, HT, and eGFR<br>3: smoking, dyslipidemia, HDL-C	UA: 1.30 (1.13–1.46) p < 0.001<br>1: 1.09 (0.94–1.26) p = 0.236<br><br>UA: 1.52 (1.27–1.83) p < 0.001<br>1: 1.39 (1.13–1.70) p = 0.001<br>2: 1.29 (1.05–1.60) p = 0.015<br>3: 1.52 (1.27–1.82) p < 0.001	[92]
			Carotid stenosis patients			
2016	264	5.3 years	Vascular mortality (MI or stroke)	Age and eGFR	UA: 1.81 (1.29–2.53) p = 0.001<br>Adj: 0.91–2.08) p = 0.129	[93]
			Hemodialysis patients			
2015	235	4 years	All-cause mortality<br><br>Cardiovascular mortality or hospitalization	Prealbumin, race, and diabetes	UA: 0.61 (0.38–0.97) p = 0.04<br>Adj: 1.14 (0.67–1.93) p = 0.62<br><br>UA: 0.71 (0.32–1.59) p = 0.40<br>Adj: 0.92 (0.40–2.10) p = 0.84	[94]
2020	252	6.1	All-cause mortality<br><br>Cardiovascular mortality	Age, SBP, CHD, diabetes, cerebral infarction, cerebral hemorrhage, gout, calcium supplement, active vitamin D, albumin, prealbumin, hemoglobin, iron, hs-CRP	UA: 1.12 (1.07–1.18) p < 0.001<br>Adj: 1.14 (1.08–1.21) p < 0.001<br><br>UA: 1.13 (1.03–1.24) p = 0.01<br>Adj: 1.18 (1.07–1.29) p = 0.001	[95]

Adj: adjusted; ANGPTL2: angiopoietin like 2; BNP: brain natriuretic peptide; BMI: body mass index; CAD: coronary artery disease; CHD: coronary heart disease; COPD: chronic

obstructive pulmonary disease; CVD: cardiovascular disease; eGFR: estimated glomerular filtration rate; HbA1c: hemoglobin A 1 c; HDL-C: high density lipoprotein cholesterol; HF: heart failure; HR: hazard-ratio; hsCRP: high sensitivity C reactive protein; HT: hypertension; LDL-C: low density lipoprotein cholesterol; MACE: major adverse cardiovascular event; MI: myocardial infarction; NT-proBNP: N-terminal pro b-type natriuretic peptide; NYHA: New York Heart Association; PAD: peripheral artery disease; SBP: systolic blood pressure; sTNFR1: soluble tumor necrosis factor receptor-1; TG: triglycerides; T2D: type 2 diabetes; UA: unadjusted; uACR: urine albumin-creatinine ratio; UAER: urinary albumin excretion rate.
